# How outbreaks in different settings contribute to the transmission of SARS-CoV-2 in the era of “living with COVID”: the case of Japan

**DOI:** 10.1016/j.lanwpc.2023.100685

**Published:** 2023-01-14

**Authors:** Char Leung, Li Su, Munehito Machida

**Affiliations:** a Department of Population Health Sciences, University of Leicester, Leicester, UK; b MRC Biostatistics Unit, School of Clinical Medicine, University of Cambridge, Cambridge, UK; c Department of International Health and Collaboration, National Institute of Public Health, Saitama, Japan

COVID-19 continues to impact different parts of the world. In countries where the “living with COVID” strategy is adopted and endemic is likely to be declared, radical public health measures, such as lockdowns, have been and will be removed. However, the transmission of SARS-CoV-2 persists and waves continue to emerge, putting pressures on the healthcare system. Because the majority of the population have been immunised, the number of the vaccinated and recovered may not be informative measures to study transmission dynamics. Instead, it is of great public health importance to understand how outbreaks in different settings contribute to the transmission of the virus differently. This is particularly true for Japan since, rather than eradicating COVID-19, reducing the number of outbreaks and maintaining the medical system while normalizing social interactions are the main goals of the country's policy on COVID-19.^1^

We demonstrated that outbreaks in certain settings contributed significantly to more confirmed cases than other settings did, using the publicly available nationwide data published by the Ministry of Health, Labour and Welfare^2^ and the Cabinet Secretariat^3^ of the Japanese Government. We collected the weekly data of the number of confirmed cases, the number of outbreaks in different settings, including medical institutions, care homes (for elderly, children, and individuals with disabilities), restaurants, sports facilities, schools and companies, and the ratio between the number of people at outdoor major points/crowds in Japan relative to that before the pandemic (to account for the impact of public health measures). Using negative binomial regression, we regressed the number of confirmed cases on the number of outbreaks in each type of settings (exposure variables), adjusting for the flow of people in public places and the number of confirmed cases in previous weeks. Details of data collection procedures, statistical analysis, and model diagnostics can be found in the Supplementary Materials.

The data were downloaded from the web pages on 10th November 2022. Because of data availability, we included the weekly data dated between 1st January 2021 and 30th October 2022, totalling 95 observations that described 38,516 outbreaks and 27,156,511 confirmed cases. 12,515 (32.5%) outbreaks occurred in elderly homes and 8247 (21.4%) in schools. Other settings accounted for less than 13% of all outbreaks. Results of the multivariable negative binomial regression model are shown in Table 1. The outbreaks at medical institutions (p < 0.001), sports facilities (p < 0.001), care homes for disabilities (p = 0.035), and companies (p < 0.001) were found to be significantly associated (at 5% level) with the number of confirmed cases. An additional outbreak in a medical institution, sports facility, care homes for disabilities, and a company was associated with 2.0%, 8.0%, 2.2%, and 1.3% increase in the number of confirmed cases, respectively.

**Table 1:** Results of negative binomial regression.

Dependent variable: Number of confirmed cases (weekly)		
Setting	Incidence rate ratio (95% confidence interval)	p
Medical institutions	1.020 (1.010,1.030)	<0.001
Care home (Disabilities)	1.022 (1.002,1.044)	0.035
Sports facilities	1.080 (1.042,1.119)	<0.001
Companies	1.013 (1.006,1.021)	<0.001
Flow of people at 8am	1.027 (1.003,1.051)	0.030
Lag 1	−2.434 × 10^−6^ (−5.458 × 10^−6^, −5.902 × 10^−7^)^a^	0.115
Lag 2	−2.977 × 10^−6^ (−7.217 × 10^−6^, −1.263 × 10^−6^)^a^	0.169
Lag 3	5.086 × 10^−6^ (−2.624 × 10^−6^, −7.459 × 10^−6^)^a^	<0.001

a Log of incidence rate ratio.

Surprisingly, while elderly homes accounted for most of the outbreaks, they did not make significant contribution to the number of confirmed cases, suggesting effective measures in reducing the transmission of the virus. Instead, outbreaks in other settings, sports facilities in particular, contributed significantly to the transmission. Although no spectators were allowed in the Summer Olympics in Tokyo, other sports events were permitted to have at most 5000 spectators, or 50% of the venue capacity.

Our analysis can inform public health policies on sporadic and more targeted measures. First, the difference in the impact on the number of confirmed cases between settings, suggesting that public health policies should aim beyond reducing the number of outbreaks. That is, more effective measures should be implemented to isolate these facilities from infection and to curb the transmission within these facilities.

Second, although the model did not show that outbreaks in elderly homes made significant contribution to the number of confirmed cases, effective infection control should remain in elderly homes. Moreover, it might be due to confounding. It is possible that cases originated in elderly homes led to hospital outbreaks upon hospitalisation. In Japan, infected residents of elderly homes are admitted to long-term care wards with different management and infection control.

Third, the difference in the impact on disease dynamics between settings might be attributed to heterogeneous compliance among lockdowns. Unlike the large scale lockdowns in other countries, sporadic lockdowns have been used by the Japanese Government for infection control.^4^ However, the effectiveness of each lockdown largely depends on the voluntary compliance of the public. Prefectural governors were given authority to request quarantine from the public but without penalties for non-compliance. One exception was the financial punishment imposed on businesses that fail to comply with requests to suspend operations.^4^ However, it is rarely enforced.

In summary, we demonstrated that outbreaks in different settings contributed differently to the transmission of SARS-CoV-2. Certain settings with a lower number of outbreaks were found to make more significant contribution to the number of confirmed cases.

## Availability of data and material

Data available upon reasonable request.

## Contributors

CL: study design, literature review, data collection, data analysis, manuscript drafting; LS: data analysis, interpretation of data; MM: interpretation of data.

## Ethics approval

Not required in the UK and Japan. Publicly available data were used.

## Declaration of interests

None.
