# Supplementary material for: How outbreaks in different settings contribute to the transmission of SARS-CoV-2 in the era of “living with COVID”: the case of Japan
Source: Lancet Reg Health West Pac. 2023 Jan 14;31:100685. doi: 10.1016/j.lanwpc.2023.100685 (PMC9839941; doi:10.1016/j.lanwpc.2023.100685)
Supplement: Supplementary Materials [file mmc1.docx]

**Supplementary Materials**

**Data collection**

The data of newly confirmed cases and outbreaks were gathered from the web page managed by the Ministry of Health, Labour and Welfare of the Japanese Government and are available at https://covid19.mhlw.go.jp/extensions/public/en/index.html. Data are collected daily by the Japanese Government from local governments and HER-SYS, a nationwide database for managing information of confirmed and suspected cases, and their close contacts. The number of outbreaks by settings were reported on a weekly basis while the number of newly confirmed cases were reported on a daily basis. Therefore, our analysis was performed on weekly data.

The data of the flow of people were gathered from the web page managed by Cabinet Secretariat of the Japanese Government and are available at https://corona.go.jp/dashboard/. The number of people were recorded at major points in each prefecture and crowds in entertainment districts at 8am, 3pm, and 9pm. The number of mobile phones was used to determine the number of people in crowds in entertainment districts. Used in our analysis was the ratio between the number of people recorded at major points/crowds of a specific week and the peak value of that in 2019.

Note that, apart from compiling daily data to weekly data, all data used in the analysis were downloaded from the abovementioned web pages without regrouping, recoding, and any other manipulations.

**Statistical analysis**

As in conventional negative binomial regression, the number of newly confirmed cases, $Y_{t}$, given the information up to time $t-1$, $\mathcal{F}_{t-1}$, has a negative binomial distribution. With the logarithmic link function and hence the lags of the log-transformed number of confirmed cases, the model is given by,

$$Y_{t}|\mathcal{F}_{t-1}\sim\mathrm{NegBinomial}(\lambda_{t},\phi)$$

$$\ln\left( \lambda_{t} \right)=\beta_{0}+\boldsymbol{\beta}^{T}\boldsymbol{X}_{t}+\boldsymbol{\gamma}^{T}\boldsymbol{Z}_{t}+\sum_{i=1}^{T} \alpha_{i}ln(Y_{t-i})$$

where $\phi$is the dispersion parameter; the vector $\boldsymbol{X}_{t}$ denotes the independent variables representing the number of outbreaks in different settings (i.e. the exposure variable); and $\boldsymbol{Z}_{t}$ denotes information of the flow of people in public places. The lags of the log-transformed number of confirmed cases,$ln(Y_{t})$, were used to account for the time-dependence^1^.

The partial autocorrelation function (PACF) was used to determine the lag order of $ln(Y_{t})$ then the choice of $\boldsymbol{X}_{t}$ and $\boldsymbol{Z}_{t}$ were determined by the backward stepwise regression procedure with p-value of 10% as the threshold. Despite being exposure variables, the choice of $\boldsymbol{X}_{t}$ was determined by the procedure because the number of outbreaks in different settings might be competing. The multivariable model was then constructed.

For model diagnostics for the multivariable model, the linearity assumption was assessed by plotting the fitted values of the model against each continuous variable and the corresponding local polynomial regression. Polynomials terms were included if the assumption was violated. The assumption of the conditional mean not equal to the conditional variance was assessed by the likelihood ratio test.

Statistical software R Version 4.2.1 and package *MASS* were used for all computations. P-value of 5% was deemed statistically significant.

**Results**

As shown in Figure S1 below, the PACF indicated the lag order of 3 (95% confidence intervals are represented by the dotted lines).

**Figure S1**. Partial autocorrelation function of the number of confirmed cases


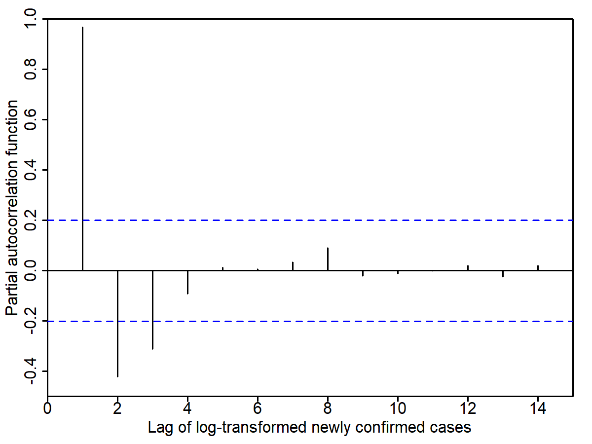


Results of univariable negative binomial regression of each variable not adjusted for lagged values of confirmed cases are shown in Table S1 below. The McFadden pseudo-R^2^ was defined as one minus the ratio between residual deviance and null deviance.

**Table S1**. Results of univariable negative binomial regression

| Dependent variable: Number of confirmed cases (weekly) | | | |
| --- | --- | --- | --- |
|  | Incidence rate ratio  (95% confidence interval) | AIC | Pseudo-R2 |
| Medical institutions | 1.023 (1.011,1.036) | 2316.332 | 0.591 |
| Care home (Elderly) | 1.004 (1.000,1.008) | 2326.562 | 0.550 |
| Care home (Children) | 1.014 (1.010,1.020) | 2295.956 | 0.663 |
| Care home (Disabilities) | 1.078 (1.045,1.114) | 2306.484 | 0.627 |
| Restaurants | 1.021 (1.007,1.037) | 2321.831 | 0.569 |
| Sports facilities | 1.130 (1.087,1.179) | 2293.448 | 0.671 |
| Schools | 1.008 (1.006,1.011) | 2290.299 | 0.681 |
| Companies | 1.018 (1.011,1.026) | 2305.516 | 0.631 |
| Others | 1.069 (1.027,1.120) | 2318.595 | 0.582 |
| Flow of people at 8am | 1.014 (0.985,1.043) | 2329.831 | 0.536 |
| Flow of people at 3pm | 1.003 (0.963,1.044) | 2330.683 | 0.532 |
| Flow of people at 9pm | 1.007 (0.988,1.027) | 2330.200 | 0.535 |

Results of multivariable negative binomial regression is shown in Table 1. The dispersion parameter was estimated to be 1.747 (95% CI 1.282-2.212). The pseudo-R^2^ and AIC were reported to be 0.7814 and 2259.7, respectively.

**Model diagnostics**

Plots of the fitted values against each continuous variable are shown in Figure S2 below. Based on the 95% prediction intervals (shaded areas), there is no sufficient evidence for violations of the non-linearity assumption.

**Figure S2**. Plots of fitted values against relative flow of people at different times


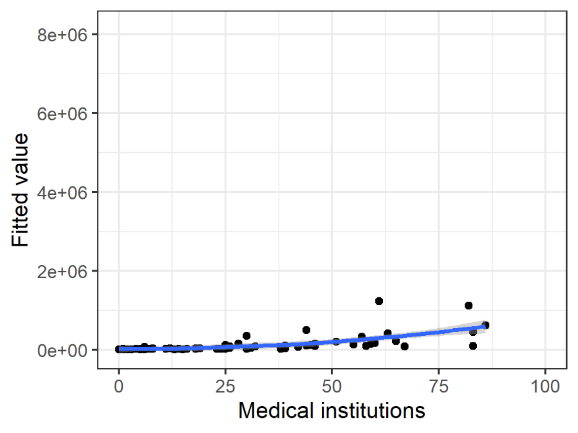

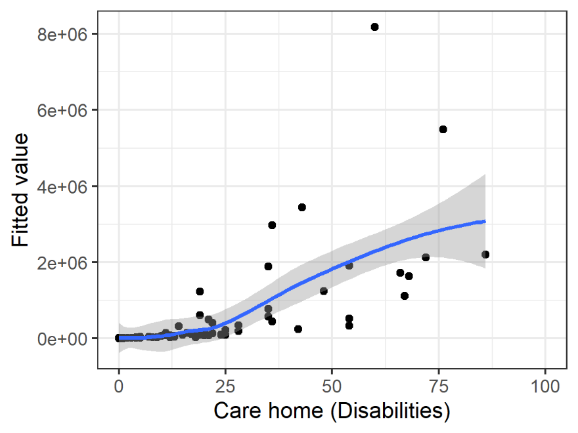


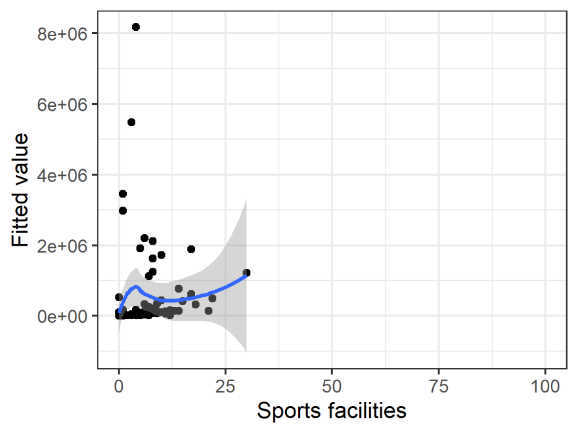

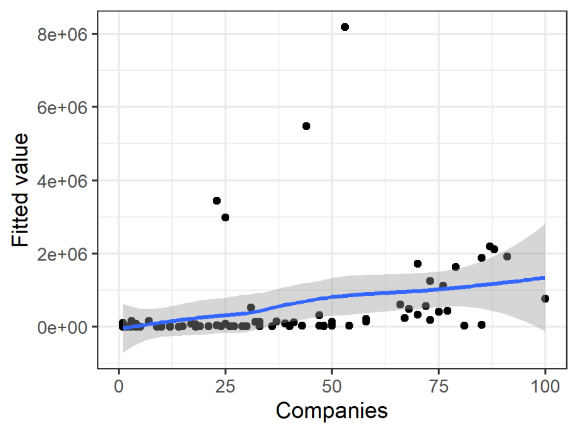


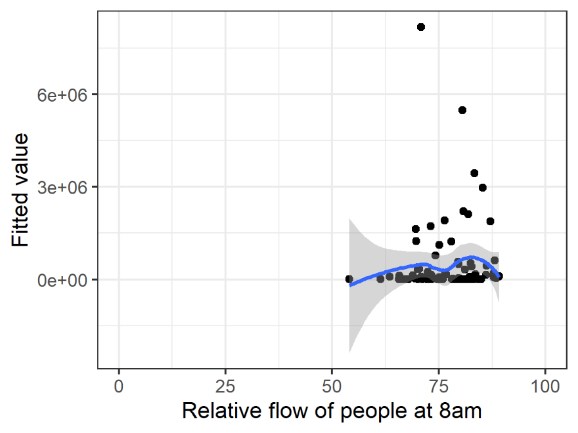


For the assumption of the conditional mean not equal to the conditional variance, the p-value of the Chi-squared test statistic was reported to be <0.001, meaning that the assumption is met.

**Reference**

1. Liboschik T, Fokianos K, Fried R. tscount: An R package for analysis of count time series following generalized linear models. J Stat Softw. 2017;82(5):1–51. 10.18637/jss.v082.i05
